# The Characteristics of TCM Clinical Trials: A Systematic Review of ClinicalTrials.gov

**DOI:** 10.1155/2017/9461415

**Published:** 2017-08-24

**Authors:** Junchao Chen, Jihan Huang, Jordan V. Li, Yinghua Lv, Yingchun He, Qingshan Zheng

**Affiliations:** ^1^Center for Drug Clinical Research, Shanghai University of Traditional Chinese Medicine, Shanghai 201203, China; ^2^Medical Science & Computing, LLC, Rockville, MD 20852, USA

## Abstract

**Objective:**

The aim of this review is to characterize current status of global TCM clinical trials registered in ClinicalTrials.gov.

**Methods:**

We examined all the trials registered within ClinicalTrials.gov up to 25 September 2015, focusing on study interventions to identify TCM-related trials, and extracted 1,270 TCM trials from the data set.

**Results:**

Overall, 691 (54.4%) trials were acupuncture, and 454 (35.8%) trials were herbal medicines. Differences in TCM trial intervention types were also evident among the specific therapeutic areas. Among all trials, 55.7% that were small studies enrolled <100 subjects, and only 8.7% of completed studies had reported results of trials. As for the location, the United States was second to China in conducting the most TCM trials.

**Conclusion:**

This review is the first snapshot of the landscape of TCM clinical trials registered in ClinicalTrials.gov, providing the basis for treatment and prevention of diseases within TCM and offering useful information that will guide future research on TCM.

## 1. Introduction

Traditional Chinese Medicine (TCM) originated in ancient China and has evolved over thousands of years [1]. TCM is now gaining popular interest worldwide and is practiced by more than 140 countries [2], which is primarily used as a complementary health approach [3]. TCM practitioners employ herbal medicines, acupuncture, and various mind and body practices (i.e., tai chi and qigong), to treat or prevent health problems. In recent years, substantial shifts in TCM use have occurred in those countries and, especially, for some disease fail to have a good efficacy with conventional therapies. As clinical trials are considered the gold standard for evaluating the safety and efficacy of therapeutics and generating evidence-based knowledge in medicine [4], growing studies have been conducted to exam the scientific evidence of TCM [5–8]. However, there are limited data regarding the current status of the TCM clinical trial enterprise.

In order to improve the transparency and accessibility to the information of clinical trials, trial registration is being required by the International Committee of Medical Journal Editors (ICMJE) [9]. Several institutions and groups have established publicly available clinical trial registries, and ClinicalTrials.gov is the largest international clinical trials registry, contains approximately 200,000 trials registered from 174 countries [10], which provides a unique opportunity to take a snapshot of global TCM clinical trials. In addition, the Clinical Trials Transformation Initiative (CTTI), established by the FDA and Duke University with over 60 institutional partners, is engaged in continuing to improve the public interface for the use of aggregate data in the ClinicalTrials.gov database [11]. Due to the fact that the detailed information on past and present clinical trials can be obtained using aggregate data of the ClinicalTrials.gov, usually there will be even more details than the reported ones in the eventual peer-reviewed publication.

The purpose of the current analysis is to describe the TCM trial portfolio using ClinicalTrials.gov data, including trial attributes, enrollment, study design, therapeutic area, location, and sponsor. Secondary aims include comparing the relationship between TCM interventions and medical conditions and describing trends in TCM trials over time.

## 2. Methods

### 2.1. Data Source

A data set of 199,269 clinical studies registered at ClinicalTrials.gov was downloaded on September 27, 2015. The data set was locked, and a relational database (SAS CPORT transport, version 9.4) was subsequently designed to facilitate analysis [12]. Details of this resource, data definitions, and data dictionaries are available at the Clinical Trials Transformation Initiative website [13].

### 2.2. Study Selection

The TCM clinical trials data set was restricted to interventional study types registered in the larger data set up to September 25, 2015 (*n* = 156,380 of 199,269). ClinicalTrials.gov defines interventional studies as those in which an investigator assigns an intervention based on a protocol. Noninterventional studies (i.e., observational or cohort studies) were eliminated to minimize bias related to the lack of reporting requirements for these studies. In order to identify trials that were potentially relevant to TCM, we focused on three trial characteristics: title, brief description, and intervention, and we chose “Chinese Medicine”, “TCM”, “Chinese Herbal”, “Acupuncture”, “Moxibustion”, “Gua Sha” “Cupping”, “Tai chi”, “Qi gong”, and so forth as conditional terms (see Item S1 in Supplementary Material available online at https://doi.org/10.1155/2017/9461415). Using a computer-based search, we identified 1,304 studies with at least one TCM-relevant term in the title, brief description, conditions, interventions, or locations. The authors (Junchao Chen and Jihan Huang) then manually reviewed each study (brief title, key words, interventions, MeSH terms, and if necessary, and the full ClinicalTrials.gov record) to determine relevance to TCM. In total, 34 studies were excluded as irrelevant to TCM, leaving a final dataset of 1,270 studies (see Supplementary Material Diagram S1).

### 2.3. Data Collection and Analysis

As previously described, trial data are self-reported by trial sponsors or investigators by using a web-based system. Each record contains a set of data elements describing the study's purpose, recruitment/enrollment, design, eligibility criteria, location, sponsor, and other protocol information; standard definitions are used, although not all fields are mandatory [14]. All the data elements of 1,270 TCM studies were fully extracted from the parent data set (*n* = 156,380). The primary therapeutic area of TCM studies was categorized by searching for the terms provided in the “conditions” data field in the ICD-10 online directory [15], and further manual classification of the “lead sponsor” by the country was performed as well. Trial characteristics were described by using standard summary statistics. Categorical variables were reported as proportions and continuous variables as medians and ranges. Due to the descriptive nature of the study, formal statistical comparisons were not made.

## 3. Results

### 3.1. Trial General and Design Characteristics

By September 25, 2015, a total of 1,270 TCM interventional trials were registered at ClinicalTrials.gov, and 3 subsets of TCM interventional trials (October 2000–September 2005, October 2005–September 2010, and October 2010–September 2015) are shown in Table 1. The average number of TCM trials registered over time was 6 trials per month from October 2005 to September 2010, and 13 trials per month from October 2010 to September 2015. Overall, 442 (34.8%) studies were in the process of recruiting and 574 (45.2%) studies were already completed. Among the completed studies, only 50 (8.7%) studies had reported results of trial on ClinicalTrials.gov. And 76.4% of TCM trials evaluated disease treatment versus 7.6% preventive therapy; 50.2% of TCM trials have DMC versus 34.8% without DMC. Most TCM studies included both male and female participants (82.2%).

Table 2 summarized the trial design characteristics for all TCM interventional trials. There were more procedure/device trials (51.7%) than drug trials (34.6%), but the drug clinical trials were increased over the 2 periods: from 21.1% in October 2000–September 2005 to 38.1% in October 2010–September 2015. Most TCM trials (55.7%) were small studies, enrolling <100 subjects, although some of the trials had an anticipated enrollment of 500 or more participants (8%). The median number of participants per trial was 90 (IQR, 45–200). A substantial proportion of TCM studies were randomized (86.5%) and double-blinded (82.2%). The majority of TCM trials were 2 arms (56.6%), and more trials included an active comparator arm (49.4%) than placebo or sham comparator arm (25.1% and 15.6%, resp.). Comparing 2 recent subsets of TCM interventional trials (October 2005–September 2010 and October 2010–September 2015), the not reported either enrollment number or type (anticipated or actual) decreased from 1.0% to 0.4%; not reported randomization increased from 6.8% to 8.5%; not reported number of arms decreased from 10.9% to 0.4%; and not reported comparator decreased from 16.2% to 0.1%.

### 3.2. Intervention Type and Therapeutic Area

**Figure 1:**
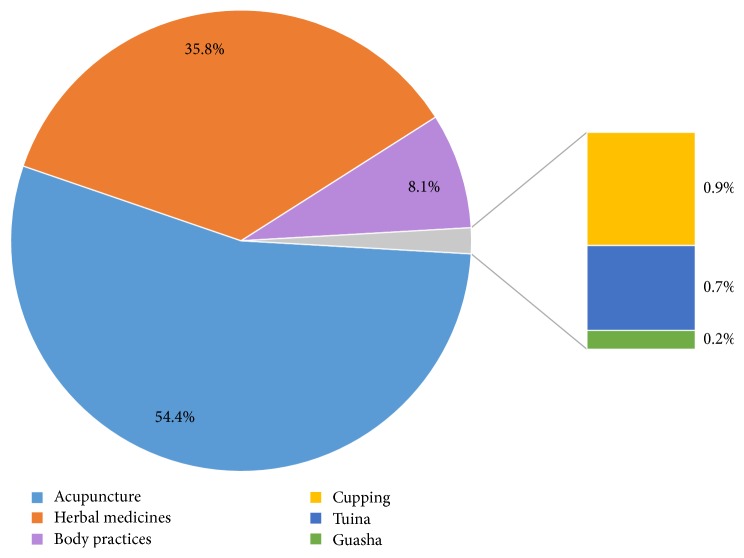
Types of interventions in TCM studies.

**Figure 2:**
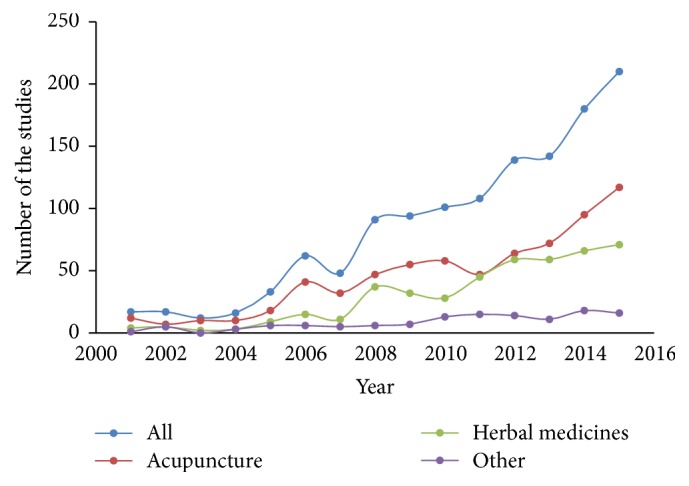
The trend of registered TCM trials on ClinicalTrials.gov.

There is a diverse range of therapeutic methods applied by TCM practitioners. All the TCM trials were manually classified according to the following TCM interventions: acupuncture, herbal medicines, mind and body practices (including taichi and qigong), cupping, tuina, and gua sha (Figure 1). As expected, the majority of TCM studies were acupuncture trials and herbal medicines trials (54.4% and 35.8%, resp.), compared with 8.1% for mind and body practices trials and 0.9% for cupping trials.  Figure 2 shows the trend of registered TCM interventional trials classified based on the types of TCM interventions used in the studies. In the last 15 years, the growth rate of acupuncture trials is higher than that of herbal medicines trials.

**Figure 3:**
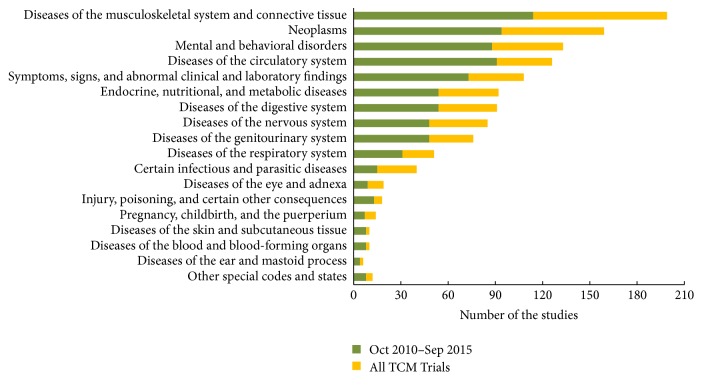
The rank of therapeutic areas by ICD-10.

The therapeutic area of all the intervention TCM trials was manually sorted by medical conditions coded with ICD-10, and the distribution of therapeutic area is presented in Figure 3. There were 21 (1.7%) trials that did not list an ICD-10 coded condition (e.g., healthy subjects). The top 5 therapeutic areas were diseases of the musculoskeletal system and connective tissue (15.9%), neoplasms (12.7%), mental and behavioral disorders (10.6%), diseases of the circulatory system (10.1%), and symptoms, signs, and abnormal clinical and laboratory findings (8.6%). Overall, 58% TCM trials were focus on the top 5 therapeutic areas. The distribution of disease-specific trials in October 2010–September 2015 is almost similar to all TCM interventional trials.

**Figure 4:**
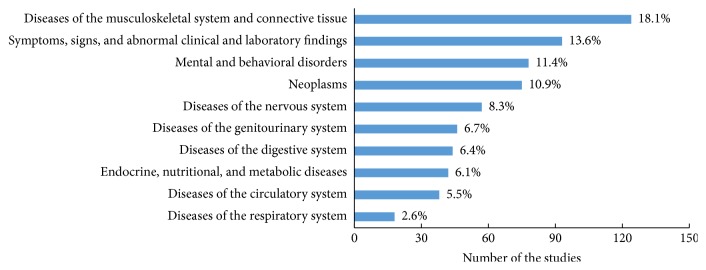
Top 10 therapeutic areas of acupuncture studies.

**Figure 5:**
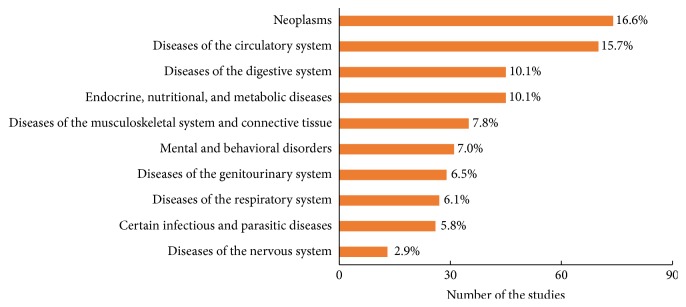
Top 10 therapeutic areas of herbal medicine studies.

Differences in TCM trial intervention types were also evident among the rank of therapeutic areas. As for acupuncture studies (Figure 4), the primary therapeutic area was diseases of the musculoskeletal system and connective tissue (18.1%), followed by symptoms, signs, and abnormal clinical and laboratory findings (13.6%). As for herbal medicine studies (Figure 5), the primary therapeutic area was neoplasms (16.6%), followed by diseases of the circulatory system (15.7%). However, there is a significant overlap among the top 10 therapeutic areas between acupuncture studies and herbal medicine studies regardless of the ranking.

### 3.3. Trial Location and Sponsor

Trial location and sponsor characteristics for all TCM interventional trials are displayed in Table 3. Overall, 52.8% of TCM trials had study sites in Asia-Pacific region versus 28.5% in North America and 15.4% in Europe. Most TCM trials were sponsored by academic institutions/medical centers (88.5%), followed by the industry (5.0%). The number of TCM studies for which the NIH was listed as the lead sponsor decreased significantly from 46 (48.4%) in the early period to 2 (0.3%) in the later period. In contrast, the percentage of trials where the lead sponsors were from academic institutions/medical centers increased from 45.3% to 92.0%.

Further manual classification of the lead sponsor by country was listed with top 10. There is no surprise that most lead sponsors of TCM trials were from China (41.5%), and the United States was second to China (28.3%); the proportion of other countries in top 10 was all less than 5%. Among all the TCM trials sponsored by the United States (*n* = 360), 70% of those trials were acupuncture trials, and the mind and body practices trials and herbal medicines trials were 18% and 13%, respectively. By comparing 2 recent periods, October 2005–September 2010 and October 2010–September 2015, there is a slight increase of TCM trials in Brazil and Spain. As for the United States, although the number of total TCM trials was approximately equivalent during those two periods, the proportion decreased from 35.4% to 18.5%.

## 4. Discussion

This analysis provides the first snapshot of the landscape of TCM clinical trials registered on ClinicalTrials.gov, whose results provide the basis for treatment and prevention of diseases within TCM, as well as the characteristics of trials design, location, and sponsor. From this review of interventional clinical trials of TCM, several noteworthy observations emerge.

We found that TCM trials were few and small in scale, majority interventional studies (55.7%) in ClinicalTrials.gov typically enrolling 100 or fewer patients. Usually, small underpowered studies have a high risk of a type II error, failing to reject the null hypothesis and inappropriately concluding that a therapy or intervention is ineffective when the sample size was too small to identify a significant effect [16]. On the one hand, many trials have similar and favorable study design characteristics between different periods (i.e., rates of randomized versus nonrandomized trials, with 2 treatment arms). On the other hand, TCM trials were also more likely to have a number of unfavorable study design characteristics, such as only half of the studies with active comparator or data monitoring committees. These findings raise concerns that well-designed trials are needed, and it also highlights the need for improvements in monitoring.

We also found that a significant number of TCM trials did not submit study results after completion of the study. This finding supports previous observations that there is low compliance of result reporting at ClinicalTrials.gov [17]. In addition, the proportion of TCM trials entered into ClinicalTrials.gov whose results are subsequently published in peer-reviewed literature is not known and requires further study. As for the missing data elements occurring for some characteristics of TCM trials, the SPIRIT (Standard Protocol Items: Recommendations for Interventional Trials) 2013 statement includes a 33-item checklist to improve the quality of clinical trial protocols [18], and perhaps greater attention to such resources may facilitate higher quality clinical trials within TCM.

We classified medical conditions using ICD-10 codes in order to examine links between TCM interventions and conditions. It shows that acupuncture trials were significantly more likely to focus on the diseases of the musculoskeletal system and connective tissue, such as low back pain and osteoarthritis, while the herbal medicines trials were significantly more likely to focus on neoplasms and diseases of the circulatory system, such as cardiovascular and cerebrovascular disease, or as combination chemotherapy in cancer treatment. Some prior reviews have reported that TCM as an adjunctive treatment can boost immunity and alleviate negative side effects experienced during chemotherapy and radiation therapy [19, 20]. Overall, almost 60% of TCM trials focus on five therapeutic areas. This finding suggests that the therapeutic benefits of TCM interventions are disease-targeted and disease-specific, which reflect in the research field of TCM clinical trials.

Our study shows that more and more TCM trials have been registered on the ClinicalTrials.gov over time, especially from China. It indicates that the ClinicalTrials.gov is a popular clinical trials registry platform for Chinese investigators or researchers as well. Although the growth rate of TCM trials in the United States has been without remarkable rise, the United States remains as the country conducting the most number of TCM clinical trials second only to China. This may likely due to TCM becoming one of the leading alternative medicines practiced in the United States [21]. The distribution of therapeutic areas of United States sponsored TCM trials was similar to all TCM interventional trials, and the top five specific diseases, subcategory of therapeutic area, were malignant neoplasms, general symptoms and signs, dorsopathies, arthropathies, and neurotic, stress-related, and somatoform disorders.

It should be noted that the data on “lead sponsor” collected in ClinicalTrials.gov represents the primary organization that oversees implementation of the study and may not necessarily represent the source of funding for the study [22]. Therefore the lead sponsor of NIH on TCM trials was decreased over time which does not mean that the funding from NIH was decreased, because the lead sponsor of academic institutions/medical centers in United States was increased, and some of the academic institutions/medical centers are likely to get the fund from NIH. It was conservatively estimated that the funding of scientific research on TCM is about $40 million annually in United States [23]. However, the majority of TCM trials sponsored by the Unite States are acupuncture trials, and herbal medicine trials are in the minority.

Although this study is the first to show specifically characteristics of TCM trials within the ClinicalTrials.gov, it is not without limitations. ClinicalTrials.gov does not include all TCM trials performed worldwide, such as partial TCM trials registered on the Chinese Clinical Trial Register (ChiCTR) [24]. We choose to focus on ClinicalTrials.gov because of the tools available for its characterization, notably the AACT database facilitated by the CTTI [11]. In addition, we mainly aim to analyze the TCM trials in other countries other than China, such as the United States. As this publically available database is updated and improved, it will continue to provide transparency regarding the type, design, distribution, and funding of TCM clinical trials. However, it is unclear whether these studies will be of sufficient quality to meet the medical needs of the growing population for TCM in worldwide.

## 5. Conclusion

Based on the data collected from the ClinicalTrials.gov, our study reveals that the content of TCM studies is dominated by small clinical trials and points out that better trials are needed. This review also provides useful information that will guide future research on TCM; in particular, our comprehensive analyses specific to certain diseases or therapeutic area may be helpful to stakeholders, including investigators, academic centers, and industry, in informing future decisions regarding the conduct of TCM trials. Given the deficit in evidence to support decisions in TCM clinical practice guideline, our analysis highlights the need for improvement in completeness of TCM study results.

## Supplementary Material

Item S1: Conditional terms specific for TCM; Diagram S1: Flow Diagram Interventional of TCM Trials.

## Figures and Tables

**Table 1:** Characteristics of TCM interventional studies.

Parameter	Number/total number (%)			
	All trials (*N* = 1270)	Oct 2000–Sep 2005 (*N* = 95)	Oct 2005–Sep 2010 (*N* = 396)	Oct 2010–Sep 2015 (*N* = 779)
*Primary purpose, N*				
Treatment	970/1270 (76.4)	77/95 (81.1)	305/396 (77.0)	588/779 (75.5)
Prevention	96/1270 (7.6)	3/95 (3.2)	30/396 (7.6)	63/779 (8.1)
Other purpose	168/1270 (13.2)	11/95 (11.6)	50/396 (12.6)	107/779 (13.7)
Missing	36/1270 (2.8)	4/95 (4.2)	11/396 (2.8)	21/779 (2.7)
*Phase, N*				
Phase 0	18/1270 (1.4)	1/95 (1.1)	3/396 (0.8)	14/779 (1.8)
Phase 1	67/1270 (5.3)	12/95 (12.6)	23/396 (5.8)	32/779 (4.1)
Phase 1/phase 2	61/1270 (4.8)	10/95 (10.5)	24/396 (6.1)	27/779 (3.5)
Phase 2	203/1270 (16.0)	28/95 (29.5)	71/396 (17.9)	104/779 (13.4)
Phase 2/phase 3	62/1270 (4.9)	3/95 (3.2)	22/396 (5.6)	37/779 (4.7)
Phase 3	131/1270 (10.3)	22/95 (23.2)	44/396 (11.1)	65/779 (8.3)
Phase 4	101/1270 (8.0)	1/95 (1.1)	23/396 (5.8)	77/779 (9.9)
Missing	627/1270 (49.4)	18/95 (18.9)	186/396 (47.0)	423/779 (54.3)
*Sex/age, N*				
Female	206/1270 (16.2)	17/95 (17.9)	72/396 (18.2)	117/779 (15.0)
Male	20/1270 (1.6)	1/95 (1.1)	6/396 (1.5)	13/779 (1.7)
Both	1044/1270 (82.2)	77/95 (81.1)	318/396 (80.3)	649/779 (83.3)
*Overall status, N*				
Not yet recruiting	96/1270 (7.6)	1/95 (1.1)	9/396 (2.3)	86/779 (11.0)
Recruiting	442/1270 (34.8)	3/95 (3.2)	71/396 (17.9)	368/779 (47.2)
Active, not recruiting	76/1270 (6.0)	1/95 (1.1)	26/396 (6.6)	49/779 (6.3)
Completed	574/1270 (45.2)	81/95 (85.3)	260/396 (65.7)	233/779 (29.9)
Terminated	40/1270 (3.1)	7/95 (7.4)	20/396 (5.1)	13/779 (1.7)
Suspended	6/1270 (0.5)	1/95 (1.1)	3/396 (0.8)	2/779 (0.3)
Withdrawn	10/1270 (0.8)	1/95 (1.1)	4/396 (1.0)	5/779 (0.6)
Enrolling by invitation	26/1270 (2.0)	0	3/396 (0.8)	23/779 (3.0)
*Study result* ^a^ *, N*				
Has result	50/574 (8.7)	2/81 (2.5)	33/260 (12.7)	15/233 (6.4)
*DMC, N*				
Has DMC	637/1270 (50.2)	10/95 (10.5)	179/396 (45.2)	448/779 (57.5)
No DMC	442/1270 (34.8)	8/95 (8.4)	151/396 (38.1)	283/779 (36.3)
DMC missing	191/1270 (15.0)	77/95 (81.1)	66/396 (16.7)	48/779 (6.2)

^a^Only including the completed studies.

**Table 2:** Trial design of TCM interventional studies.

Parameter	Number/total number (%)			
	All Trials (*N* = 1270)	Oct 2000–Sep 2005 (*N* = 95)	Oct 2005–Sep 2010 (*N* = 396)	Oct 2010–Sep 2015 (*N* = 779)
*Type of intervention, N*				
Drug	439/1270 (34.6)	20/95 (21.1)	122/396 (30.8)	297/779 (38.1)
Procedure/Device	657/1270 (51.7)	58/95 (61.1)	223/396 (56.3)	376/779 (48.3)
Behavioral	145/1270 (11.4)	15/95 (15.8)	43/396 (10.9)	87/779 (11.2)
Other intervention	29/1270 (2.3)	2/95 (2.1)	8/396 (2.0)	19/779 (2.4)
*Enrollment, N*				
Median (interquartile range)	90.0 (45.0–200.0)	90.0 (45.0–140.0)	80.0 (40.0–166.0)	96.0 (50.0–216.0)
1–100	708/1270 (55.7)	47/95 (49.5)	239/396 (60.4)	422/779 (54.2)
101–500	445/1270 (35.0)	24/95 (25.3)	129/396 (32.6)	292/779 (37.5)
501–1000	59/1270 (4.6)	4/95 (4.2)	14/396 (3.5)	41/779 (5.3)
>1000	31/1270 (2.4)	0	10/396 (2.5)	21/779 (2.7)
Missing	27/1270 (2.1)	20/95 (21.1)	4/396 (1.0)	3/779 (0.4)
*Allocation, N*				
Randomized	1099/1270 (86.5)	80/95 (84.2)	344/396 (86.9)	675/779 (86.6)
Nonrandomized	69/1270 (5.4)	6/95 (6.3)	25/396 (6.3)	38/779 (4.9)
Missing	102/1270 (8.0)	9/95 (9.5)	27/396 (6.8)	66/779 (8.5)
*Masking/blinding*				
Open	206/1270 (16.2)	17/95 (17.9)	72/396 (18.2)	117/779 (15.0)
Single-blind	20/1270 (1.6)	1/95 (1.1)	6/396 (1.5)	13/779 (1.7)
Double-blind	1044/1270 (82.2)	77/95 (81.1)	318/396 (80.3)	649/779 (83.3)
*Number of arms, N*				
1	132/1270 (10.4)	2/95 (2.1)	52/396 (13.1)	78/779 (10.0)
2	719/1270 (56.6)	10/95 (10.5)	207/396 (52.3)	502/779 (64.4)
3	226/1270 (17.8)	7/95 (7.4)	73/396 (18.4)	146/779 (18.7)
4	58/1270 (4.6)	2/95 (2.1)	13/396 (3.3)	43/779 (5.5)
≥5	15/1270 (1.2)	0	8/396 (2.0)	7/779 (0.9)
Missing	120/1270 (9.4)	74/95 (77.9)	43/396 (10.9)	3/779 (0.4)
*Comparator, N*				
Experimental	1116/1270 (87.9)	20/95 (21.1)	332/396 (83.8)	778/779 (99.9)
Active comparator	627/1270 (49.4)	11/95 (11.6)	189/396 (47.7)	427/779 (54.8)
Placebo comparator	319/1270 (25.1)	10/95 (10.5)	109/396 (27.5)	200/779 (25.7)
Sham comparator	198/1270 (15.6)	7/95 (7.4)	65/396 (16.4)	126/779 (16.2)
No intervention	197/1270 (15.5)	2/95 (2.1)	52/396 (13.1)	143/779 (18.4)
Other	71/1270 (5.6)	1/95 (1.1)	20/396 (5.1)	50/779 (6.4)
Missing	140/1270 (11.0)	75/95 (78.9)	64/396 (16.2)	1/779 (0.1)

**Table 3:** Trial location and sponsor of TCM interventional studies.

Parameter	Number/total number (%)			
	All Trials (*N* = 1270)	Oct 2000–Sep 2005 (*N* = 95)	Oct 2005–Sep 2010 (*N* = 396)	Oct 2010–Sep 2015 (*N* = 779)
*Region, N*				
Africa	8/1270 (0.6)	1/95 (1.1)	1/396 (0.3)	6/779 (0.8)
Asia-Pacific	670/1270 (52.8)	28/95 (29.5)	185/396 (46.7)	457/779 (58.7)
Central and South America	52/1270 (4.1)	5/95 (5.3)	24/396 (6.1)	23/779 (3.0)
Europe	195/1270 (15.4)	19/95 (20.0)	81/396 (20.5)	95/779 (12.2)
Middle East	28/1270 (2.2)	0	10/396 (2.5)	18/779 (2.3)
North America	362/1270 (28.5)	78/95 (82.1)	140/396 (35.4)	144/779 (18.5)
*Lead sponsor, N*				
Industry	63/1270 (5.0)	5/95 (5.3)	13/396 (3.3)	45/779 (5.8)
NIH	55/1270 (4.3)	46/95 (48.4)	7/396 (1.8)	2/779 (0.3)
US Fed (non-NIH)	28/1270 (2.2)	1/95 (1.1)	12/396 (3.0)	15/779 (1.9)
Other^a^	1124/1270 (88.5)	43/95 (45.3)	364/396 (91.9)	717/779 (92.0)
*Country of lead ponsor* ^b^ *, N*				
China^c^	527/1270 (41.5)	14/95 (14.7)	142/396 (35.9)	371/779 (47.6)
United States	360/1270 (28.3)	76/95 (80.0)	140/396 (35.4)	144/779 (18.5)
Korea	58/1270 (4.6)	1/95 (1.1)	13/396 (3.3)	44/779 (5.6)
Germany	42/1270 (3.3)	1/95 (1.1)	19/396 (4.8)	22/779 (2.8)
Canada	39/1270 (3.1)	3/95 (3.2)	17/396 (4.3)	19/779 (2.4)
Brazil	35/1270 (2.8)	2/95 (2.1)	10/396 (2.5)	23/779 (3.0)
Israel	20/1270 (1.6)	0	8/396 (2.0)	12/779 (1.5)
Spain	17/1270 (1.3)	1/95 (1.1)	3/396 (0.8)	13/779 (1.7)
United Kingdom	16/1270 (1.3)	2/95 (2.1)	7/396 (1.8)	7/779 (0.9)
Sweden	15/1270 (1.2)	0	8/396 (2.0)	7/779 (0.9)

^a^For the TCM trials, further manual classification of the “other” lead sponsor group found that this group was all composed of academic institutions/medical centers. ^b^Only listing the top 10 countries of lead sponsor. ^c^Including Taiwan and Hong Kong.
