# Supplementary material for: The Characteristics of TCM Clinical Trials: A Systematic Review of ClinicalTrials.gov
Source: Evid Based Complement Alternat Med. 2017 Aug 24;2017:9461415. doi: 10.1155/2017/9461415 (PMC5613643; doi:10.1155/2017/9461415)
Supplement: Supplementary file 1 — Item S1: Conditional terms specific for TCM; Diagram S1: Flow Diagram Interventional of TCM Trials. [file 9461415.f1.docx]

**Item S1**: Conditional terms specific for TCM

Acupuncture

Electroacupuncture

Auriculotherapy

Acupressure

Moxibustion

Traditional Chinese Medicine

Chinese Herbal Medicine

Chinese Herbals

TCM

Tai chi

Qi gong

Tui na

Cupping

Gua Sha

**Diagram S1.** Flow Diagram Interventional of TCM Trials

## Screening

## Eligibility

Trials in CT.GOV database downloaded September 27, 2015

(n = 199269)

Restrict to “interventional” registered from Sep 25 2015

(n =156380)

(n =2102)

Identified trials with conditional terms relevant to TCM
(n = 1304)

Trials included in analysis
(n = 1270)

Manual review excluded trials

(n =34)

## Identification

## Included
